# Supplementary material for: Development and Validation of a Model to Predict Severe Hospital-Acquired Acute Kidney Injury in Non-Critically Ill Patients
Source: J Clin Med. 2021 Aug 31;10(17):3959. doi: 10.3390/jcm10173959 (PMC8432169; doi:10.3390/jcm10173959)
Supplement: Supplementary file 1 [file jcm-10-03959-s001.zip › jcm-1262973-supplementary.pdf]

## SUPPLEMENTARY MATERIALS

**Table S1.** List of nephrotoxic drugs included.

| Non-steroidal anti-inflammatory drugs | Antibiotics | Antiviral agents | Antifungal agents | Immunosuppressors | Chemotherapy | Others |
|---|---|---|---|---|---|---|
| All | Vancomycin<br>Aminoglycosides: amikacin, gentamicin, netilmicin<br>Sulfadiazine | Nucleosidic inhibitors: acyclovir, adefovir, cidofovir, tenofovir, indinavir<br>Foscarnet | Amphotericin B<br>Caspofungin<br>Voriconazole | Cyclosporin<br>Tacrolimus<br>Everolimus<br>Temsirolimus<br>Immunoglobulins containing sucrose | Cisplatin,<br>Carboplatin<br>Gemcitabine<br>Ifosfamide<br>Mitramicin<br>Pemetrexed<br>Linalidomide<br>Venetoclax<br>Pentostatine<br>Imatininib,<br>Dasatininb<br>Methotrexate (high dose > 10 g/m2)<br>VEGF inhibitors<br>Ibrutinib | Mannitol<br>Lithium<br>Zoledronic acid |
